# Overcoming MRSA Antibiotic Resistance Through Losartan Repurposing with Carbon Dot–Conjugated Cerosomal Nanocarriers

**DOI:** 10.3390/pharmaceutics17111483

**Published:** 2025-11-17

**Authors:** Yasmina Elmahboub, Rofida Albash, Ahmed M. Agiba, Mariam Hassan, Haneen Waleed Mohamed, Mohamed Safwat Hassan, Roaa Mohamed Ali, Yara E. Shalabi, Hend Mahmoud Abdelaziz Omran, Ahmed Adel Alaa-Eldin, Jawaher Abdullah Alamoudi, Asmaa Saleh, Amira B. Kassem, Moaz A. Eltabeeb

**Affiliations:** 1 Department of Pharmaceutics, College of Pharmaceutical Sciences and Drug Manufacturing, Misr University for Science and Technology, Giza 12585, Egypt; yasmina.elmahboub@must.edu.eg; 2 School of Engineering and Sciences, Tecnologico de Monterrey, Monterrey 64849, Mexico; 3 Department of Microbiology and Immunology, Faculty of Pharmacy, Cairo University, Cairo 11562, Egypt; mariam.hassan@pharma.cu.edu.eg; 4 Department of Microbiology and Immunology, Faculty of Pharmacy, Galala University, New Galala City, Suez 43511, Egypt; 5 College of Pharmaceutical Sciences and Drug Manufacturing, Misr University for Science and Technology, Giza 12585, Egypt; 6 Department of Pharmaceutics, Faculty of Pharmacy, Fayoum University, Faiyum 2933051, Egypt; aam34@fayoum.edu.eg; 7 Department of Pharmaceutical Sciences, College of Pharmacy, Princess Nourah Bint Abdulrahman University, P.O. Box 84428, Riyadh 11671, Saudi Arabia; jaalamoudi@pnu.edu.sa (J.A.A.); asali@pnu.edu.sa (A.S.); 8 Clinical Pharmacy and Pharmacy Practice Department, Faculty of Pharmacy, Damanhour University, Damanhour 22514, Egypt; amira.kassem@pharm.dmu.edu.eg; 9 Department of Industrial Pharmacy, College of Pharmaceutical Sciences and Drug Manufacturing, Misr University for Science and Technology, Giza 12585, Egypt; moaz.eltabib@must.edu.eg

**Keywords:** drug repurposing, losartan potassium, MRSA, cerosomes, carbon dots

## Abstract

**Background/Objectives:** Methicillin-resistant *Staphylococcus aureus* (MRSA) presents a serious hurdle in combating antibiotic-resistant skin infections. This study aimed to repurpose losartan potassium (LOS) through its incorporation into cerosomal nanocarriers (CERs) and further functionalization with carbon dots (CDs) to enhance antibacterial efficacy. **Methods:** LOS-CERs were fabricated by the thin-film hydration method and further optimized using a D-optimal mixture design. **Results:** The optimized CERs, composed of phytantriol (20 mg), ceramide (30 mg), and CTAB (20 mg), exhibited high entrapment efficiency (97.07 ± 0.07%), a nanoscale particle size (372.50 ± 0.50 nm), and a positive zeta potential (+33.24 ± 0.04 mV). FT-IR analysis confirmed successful conjugation of CDs to CERs through surface functional interactions. Ex vivo permeation and confocal microscopy studies demonstrated that the CD-CER formulation sustained LOS release and enhanced its deposition within skin layers compared with the LOS solution. Using a murine model of MRSA USA300-induced skin infection, the CD-CER formulation achieved superior antibacterial efficacy, reducing the bacterial load by 3.85 log_10_ CFU relative to the untreated control, compared with a 3.04 log_10_ CFU reduction for the LOS solution. Histological evaluation supported improved healing in CD-CER-treated groups. **Conclusions:** Overall, CD-functionalized CERs offer a promising multifunctional nanoplatform for repurposing LOS as a topical therapeutic against MRSA-associated skin infections.

## 1. Introduction

Antibiotic-resistant bacterial infections are an escalating global health concern, posing significant health risks to individuals of all ages. In neonates, these bacterial infections often manifest as early-onset sepsis, a major contributor to morbidity and mortality during the first week of life [1]. Among resistant bacterial pathogens, methicillin-resistant *Staphylococcus aureus* (MRSA), historically regarded as a hospital-associated pathogen, has now emerged as a widespread threat in community settings. These infections are frequently severe, contribute to prolonged hospitalization, and are associated with elevated mortality rates [2]. The transmission of MRSA pathogenic bacteria mainly occurs through direct contact with infected wounds, contaminated surfaces, or transient colonization of the hands of healthcare workers. Although MRSA often persists asymptomatically on the skin or mucosal surfaces, it is an opportunistic pathogen capable of inducing a broad spectrum of serious infections, ranging from mild cutaneous lesions to severe, life-threatening illnesses, including arthritis, endocarditis, necrotizing pneumonia, septicemia, and toxic shock syndrome (TSS) [3]. The clinical burden of MRSA pathogenic bacteria has grown significantly, driven by its resistance to multiple antibiotics and its capability to form biofilms that protect it from treatment. One emerging approach to overcome these barriers is the repurposing of non-antibiotic drugs that have shown antibacterial activity in preclinical laboratory research. This strategy involves identifying new therapeutic uses for drugs already approved by regulatory authorities for other disease conditions. Given their established safety, tolerability, and pharmacokinetic profiles, repurposing offers the advantage of accelerating drug development while minimizing the potential risks involved with introducing new antimicrobials to clinical practice [4].

MRSA has the capacity to persist within host cells, making intracellular survival a major obstacle to effective treatment. The efficacy of antimicrobial therapy hinges on the drug’s ability to reach and penetrate cellular membranes, achieve adequate intracellular concentrations, and sustain therapeutic drug levels within infected tissues [5]. Drug nanocarriers have emerged as promising solutions to address this challenge, as they can improve drug delivery by enhancing cellular uptake, improving tissue retention, and providing controlled drug release [6]. By directly engaging with MRSA and gradually releasing their therapeutic payloads, nanocarriers provide prolonged antibacterial activity. One example of such advanced nanocarriers is cerosomes (CERs), vesicular nanocarrier structures composed of phospholipids and ceramides (waxy lipid molecules naturally abundant in the skin, comprising more than 50% of its total lipid content). CERs are particularly advantageous for skin-associated infections due to their high skin affinity, enhanced stability, and the capacity to fuse with cellular membranes [7,8]. Carbon dots (CDs), a class of nanomaterials known for their strong fluorescence properties, typically have a core–shell structure, with the core comprising a graphitic carbon lattice, and the shell composed of abundant functional groups or polymer chains [9]. CDs exert antibacterial activity through multiple mechanisms, making it difficult for bacteria to develop resistance, thereby highlighting their potential as promising antimicrobial agents [10]. When CERs are conjugated with CDs, renowned for their excellent biocompatibility, tunable surface chemistry, and intrinsic antibacterial activity, they confer synergistic advantages [11]. These carbon dot-functionalized cerosomes (CD-CERs) not only improve drug loading and controlled release but also enhance cellular internalization and antibacterial efficacy [12]. Collectively, this hybrid platform holds a significant promise for targeting both extracellular and intracellular MRSA in skin infections.

To date, few studies have explored the integration of drug repurposing with functionalized nanocarriers to combat MRSA. The present study differs from previous studies by combining three complementary elements: (i) the use of losartan potassium (LOS), an angiotensin II receptor blocker recently shown to exhibit antibacterial activity against MRSA [13]; (ii) the development of a hybrid multifunctional nanocarrier based on CERs functionalized with CDs to enhance stability, skin adhesion, and intrinsic antimicrobial performance; and (iii) the systematic optimization of this delivery system using a D-optimal mixture design. LOS was selected because of its dual therapeutic potential: (1) direct antibacterial effects demonstrated in our previous work [13] and (2) its known anti-inflammatory and tissue-repair properties that could synergize with the CD–CER platform to improve infection control and wound healing. This synergistic combination is expected to enhance localized antibacterial efficacy while minimizing systemic exposure, representing a novel strategy for managing drug-resistant skin and implant-associated infections.

In the current study, CD-CERs were developed as a functionalized nanocarrier for repurposed LOS to treat MRSA-associated skin infections, with the aim of optimizing skin distribution and retention of LOS and evaluating the in vivo safety through histopathological examination and the therapeutic efficacy of the proposed formulation. By applying a D-optimal mixture design, we examined the effects of four different formulation factors: type of cosurfactant (glycerol or phytantriol; X_1_), amount of cosurfactant (X_2_), amount of ceramide (X_3_), and amount of cetyltrimethylammonium bromide (CTAB; X_4_). Their effects were evaluated based on three key responses: entrapment efficiency (EE%; Y_1_), particle size (PS; Y_2_), and zeta potential (ZP; Y_3_). Ex vivo permeation analysis compared the skin distribution of LOS solution versus the optimized CD-CER formulation, while confocal microscopy tracked the deposition of fluorescein-labeled CD-CER formulation within the various layers of the skin. Subsequent to the in vitro and ex vivo analyses, in vivo investigations were conducted in a murine model to further validate the therapeutic performance of the optimized CD-CER formulation. These experiments were designed to assess not only the antibacterial efficacy against MRSA-associated skin infections but also the safety profile of the optimized CD-CER formulation under physiologically relevant conditions.

## 2. Materials and Methods

### 2.1. Materials

Losartan potassium (LOS) was kindly provided by the Egyptian International Pharmaceutical Industries Co. (Cairo, Egypt). Phospholipid (100 mg), glycerol, phytantriol, cetyltrimethylammonium bromide (CTAB), urea, citric acid anhydrous, and fluorescein diacetate (FDA) were purchased from Sigma-Aldrich (St. Louis, MO, USA). Ceramide was obtained from Evonik Röhm GmbH (Darmstadt, Germany). Methanol and chloroform were obtained from Merck KGaA (Darmstadt, Germany). All remaining chemicals and reagents used were of high analytical grade.

### 2.2. Methods

Preparation of Losartan-Loaded Cerosomes

Losartan-loaded cerosomes (LOS-CERs) were fabricated by the mechanical dispersion thin-film hydration method. The phospholipid amount maintained at a constant 100 mg to ensure consistent bilayer formation and reproducible vesicle characteristics [14]. Briefly, varying amounts of glycerol or phytantriol (as cosurfactants), CTAB surfactant, and ceramide were dissolved in 10 mL of a methanol and chloroform mixture at a ratio of 1:2 (*v*/*v*). The organic solvents were removed under reduced pressure at 60 °C using a rotary evaporator (Heidolph Instruments GmbH & Co. KG, Schwabach, Germany) set at 90 rpm for 30 min, forming a dry lipid film. This film was then hydrated with 10 mL of deionized water containing 75 mg of LOS at 60 °C. To ensure proper hydration, small glass beads were used during the 45 min of agitation. The resulting LOS-CER dispersion was kept at 4 °C until further use.

### 2.3. Characterization of Losartan-Loaded Cerosomes

#### 2.3.1. Determination of Entrapment Efficiency (EE%) and Drug Loading Capacity (LC%)

The LOS-CER dispersions were subjected to high-speed centrifugation (Sigma-3K30, Sigma Laborzentrifugen GmbH, Osterode am Harz, Germany) at 20,000 rpm for 1 h at 4 °C. The resulting supernatant was collected, appropriately diluted, and analyzed to quantify the unentrapped (free) LOS. The absorbance was measured at the wavelength of 235 nm using a UV-Vis spectrophotometer (Shimadzu UV-1650, Shimadzu Corporation, Kyoto, Japan). The entrapment efficiency (EE%) was calculated using the following equation [15].

(1) $$\mathrm{EE}\%=\left(\frac{Total\;LOS-Unentrapped\;\left(free\right)\;LOS}{Total\;LOS}\right)\;\times \;100$$

The drug loading capacity (LC%) was calculated using the following equation [16].

(2) $$\mathrm{LC}\%=\left(\frac{Entrapped\;LOS}{Total\;CERs}\right)\;\times \;100$$

#### 2.3.2. Determination of Particle Size (PS), Polydispersity Index (PDI), and Zeta Potential (ZP)

Following suitable dilution, the particle size (PS), polydispersity index (PDI), and zeta potential (ZP) of the cerosomal formulations were measured using a Zetasizer (Malvern Instruments Ltd., Worcestershire, UK). Measurements were performed in triplicate for each formulation, and the averages were recorded [16].

#### 2.3.3. D-Optimal Mixture Design and Selection of the Optimum LOS-CERs

A D-optimal mixture design was employed to examine the effects of four different formulation factors: type of cosurfactant (glycerol or phytantriol; X_1_), amount of cosurfactant (mg) (X_2_), amount of ceramide (mg) (X_3_), and amount of CTAB (mg) (X_4_) (Table 1). Each response was analyzed by analysis of variance (ANOVA) using Design-Expert^®^ software version 13 (Stat-Ease Inc., Minneapolis, MN, USA), with statistical significance set at *p* < 0.05 [15].

Formulation optimization was achieved using a composite desirability function methodology that maximized EE% (Y_1_) and ZP (Y_3_) while minimizing PS (Y_2_). The formulation with the highest desirability (nearest to 1) was re-prepared and experimentally tested to confirm the model predictions [15].

### 2.4. Synthesis of Carbon Dots

Carbon dots (CDs) were synthesized by dissolving urea and citric acid anhydrous in a 1:1 mass ratio in 10 mL of distilled water. The mixture was then subjected to microwave irradiation using a domestic microwave oven (850 W) for 5 min, during which the clear solution gradually turned yellowish-brown, indicating the formation of CDs [17]. The solution was cooled to room temperature and then diluted with 10 mL of distilled water. Unreacted materials were removed by sequential filtration through 0.45 µm and 0.22 µm nylon syringe filters (Millipore, Billerica, MA, USA). The filtered solution was further purified using a cellulosic membrane dialysis bag (MWCO: 12,000–14,000 Da) for 48 h, with the dialysis medium changed daily. Finally, the purified CDs were kept at 4 °C until further use. The synthesized CDs possessed a negative surface charge due to the presence of carboxyl and hydroxyl functional groups on their surface, which facilitated their subsequent electrostatic conjugation with the positively charged cerosomes. The UV–Vis absorption spectrum of the prepared CDs is shown in Supplementary Materials Figure S1.

### 2.5. Preparation of Carbon Dot-Functionalized Cerosomes

CD-CERs were prepared by mixing the optimized LOS-CERs with CDs in a 1:1 mass ratio under magnetic stirring for 30 min at room temperature. The electrostatic attraction between the negatively charged CDs and the positively charged cerosomal surface enabled efficient conjugation without requiring additional coupling agents (e.g., EDC/NHS or silane) [18]. The resulting mixture was then kept at 4 °C until further use.

A blank CD-CER formulation was also prepared following the same procedure, except that LOS was omitted during the hydration step. This blank formulation served as a control for physicochemical characterization to confirm the successful conjugation of CDs with the cerosomal matrix and to differentiate the spectral features of the carrier components from those of the drug-loaded formulation.

### 2.6. Fourier-Transform Infrared (FT-IR) Spectroscopy

The Shimadzu FT-IR-8400 spectrometer (Shimadzu Corporation, Kyoto, Japan) was used to record the FT-IR spectra of LOS, the optimized LOS-CER formulation, CD-CERs, and Blank (CD-CERs) at ambient temperature. For analysis, approximately 2–3 mg of each sample was mixed with potassium bromide (KBr), compressed into a single pellet, and scanned over a wavenumber range of 400–4000 cm^−1^.

### 2.7. Effect of Storage

The optimized CD-CER formulation was stored in sealed amber glass vials at 4–8 °C for 3 months. The samples were periodically inspected for any signs of phase separation or precipitate formation. After completing 3 months of storage, EE%, PS, ZP, and PDI were re-measured, and the results were statistically analyzed using a paired *t*-test in SPSS^®^ software version 22.0 (SPSS Inc., Chicago, IL, USA) [19].

### 2.8. Morphological Examination by Transmission Electron Microscopy (TEM)

The shape and structure of the optimized LOS-CER formulation and CD-CERs were examined using a transmission electron microscope (TEM) (JEM-1230, JEOL Ltd., Tokyo, Japan). A small drop of each formulation was applied onto a carbon-coated copper grid, allowed to air-dry, and then examined under the TEM for imaging [15].

### 2.9. Determination of Drug Release

Release experiments were carried out using a custom-built Franz diffusion cell with an effective diffusion area of 3.14 cm^2^ [15]. A cellulosic membrane separated the donor and receptor compartments, functioning as a controllable diffusion barrier layer. The donor compartment was charged with 1 mL of the optimized CD-CER formulation or an equivalent amount (7.5 mg) of LOS solution. The receptor compartment held 50 mL of phosphate buffer (0.1 M, pH 5.5), maintained at 37 ± 0.5 °C to simulate physiological conditions, and continuously stirred to ensure uniform mixing. At designated time points (1, 2, 3, 4, 5, 6, and 8 h), 1 mL samples were collected from the receptor compartment and immediately replaced with an equal volume of fresh phosphate buffer to maintain sink conditions. The concentration of LOS in each sample was quantified spectrophotometrically by measuring absorbance at wavelength of 235 nm using a UV-Vis spectrophotometer [15]. All measurements were carried out in triplicate, and release data are reported as mean ± standard deviation (SD). Comparative statistical analysis of release profiles was performed using ANOVA, with a *p*-value < 0.05 considered statistically significant.

### 2.10. Ex Vivo Permeation Studies

The permeation experiment was carried out using both the optimized CD-CER formulation and an equivalent LOS solution. Cylindrical diffusion tubes with an effective permeation area of 3.14 cm^2^ (corresponding to a circular surface with a 1 cm radius) were employed. One end of each tube was sealed with excised skin membrane. The other was fixed to the shaft of a USP Dissolution Apparatus I (Pharma Test PT-DT, Pharma Test GmbH, Hainburg, Germany), replacing the standard basket configuration [15]. The whole system was maintained at 37 ± 0.5 °C, and the formulations were immersed in 50 mL of phosphate buffer (0.1 M, pH 5.5) under sink conditions. At designated time points (1, 2, 3, 4, 6, 12, and 24 h), samples were collected, immediately replaced with an equal volume of fresh phosphate buffer, and analyzed by high-performance liquid chromatography (HPLC) using a Waters Alliance 2690 HPLC system equipped with a 996 PDA detector (Waters, Milford, MA, USA) according to our previously published protocol [13]. Statistical analysis was carried out using Student’s *t*-test, with a *p*-value < 0.05 considered statistically significant. At the end of the experiment, the skin tissues were removed from the diffusion tubes and rinsed with distilled water for 10 s to eliminate any adhering drug. The tissues were then cut into small pieces and sonicated in 5 mL of methanol for 30 min using a water bath sonicator to extract the deposited drug. The resulting samples were subsequently analyzed by HPLC [13].

### 2.11. Confocal Laser Scanning Microscopy (CLSM) for Skin Penetration and Distribution Studies

To investigate the distribution of the optimized CD-CER formulation within skin tissue, a fluorescently labeled form of the optimized formulation was freshly prepared. In this preparation, LOS was excluded and replaced with 10 mg of fluorescein diacetate (FDA) as a lipophilic fluorescent probe. FDA was selected due to its ability to permeate biological membranes and undergo enzymatic conversion to fluorescein, thereby enabling visualization of its penetration depth. The experimental setup for the skin was identical to that used in the release experiments [15]. The FDA-loaded formulation was applied to the surface of the skin and left undisturbed for 6 h. After the application period, longitudinal sections of the treated skin were obtained using a microtome (Cambridge Instruments Ltd., Cambridge, UK). The tissue sections were then analyzed under an inverted fluorescence microscope (Carl Zeiss GA, Zeiss, Germany) to assess the penetration and distribution of FDA throughout the skin layers. Control experiments with free FDA solutions were carried out under identical conditions for comparison.

### 2.12. In Vivo MRSA Skin Infection Model

All animal experiments and handling procedures were performed in compliance with the Guide for the Care and Use of Laboratory Animals (8^th^ edition; ILAR, Washington, DC, USA) and reviewed and approved by the Research Ethics Committee of the Faculty of Pharmacy, Cairo University (Approval No.: MI3916; 26 May 2025) [20]. The MRSA skin infection model was conducted as previously described [21,22]. 18 male BALB/c mice (8 weeks old) were obtained from a licensed local supplier and maintained under standard laboratory and environmental conditions with *ad libitum* access to food and water. Before the experimentation, the dorsal backs of mice were shaved. Each mouse was inoculated intradermally with 70 µL of MRSA USA300 suspension (9 × 10^8^ CFU/mL in sterile saline) in the lower back region. The mice were randomly assigned to three groups (*n* = 6 per group). After 48 h post-infection, once an abscess-type wound had formed, treatments were applied as follows: group 1 received topical application of the optimized CD-CER formulation (50 mg/mL), group 2 received an equivalent concentration of LOS solution (50 mg/mL), and group 3 served as the untreated control group. Each group received 100 µL of the assigned formulation topically at the infection site once daily for three days. 24 h after the final treatment, the animals were euthanized. The skin lesions were excised and homogenized in 0.5 mL of sterile saline (Daihan Scientific Co., Ltd., Republic of Korea). Homogenates were serially 10-fold diluted, plated on mannitol salt agar, and incubated at 37 °C for 24 h. Colony-forming units (CFU) were then counted, and the results were assessed and compared among the tested groups. Statistical comparisons among the tested groups were carried out using one-way ANOVA followed by Tukey’s post hoc test.

### 2.13. Histopathological Examination

Skin specimens from the infection sites of all experimental groups (control, LOS solution, and CD-CERs) were collected after the final treatment and immediately fixed in 10% neutral buffered formalin for 24 h. The fixed tissues were then trimmed, washed in running tap water, dehydrated in ascending grades of ethyl alcohol, cleared in xylene, and embedded in paraffin wax. Thin sections (4–6 µm) were prepared using a rotary microtome, mounted on glass slides, and stained with hematoxylin and eosin (H&E) according to the standard method described by Bancroft et al. [23]. The stained sections were examined under a light microscope (Leica Microsystems, Wetzlar, Germany) for the assessment of epidermal integrity, inflammatory cell infiltration, necrosis, and overall tissue architecture.

### 2.14. Statistical Analysis

Statistical analyses were performed using SPSS^®^ software version 22.0 (SPSS Inc., Chicago, IL, USA). For comparisons between two groups, either a paired *t*-test or an unpaired Student’s *t*-test were applied, as appropriate. For comparisons among more than two groups, one-way ANOVA was performed, followed by Tukey’s post hoc multiple comparison test.

## 3. Results and Discussion

### 3.1. Optimization of LOS-CERs Using D-Optimal Mixture Design

A Quality by Design (QbD) approach was employed to optimize the formulation of LOS-CERs. QbD provides a structured framework for pharmaceutical product development, enabling deeper process understanding, improved formulation efficiency, and accelerated development and production timelines. To identify the optimal levels of the formulation variables, the desirability function was applied as an effective optimization tool [24]. Model fitting indicated that EE% followed a quadratic model, whereas PS and ZP were best described by linear models. Model validity was confirmed by adequate precision, with all responses showing values exceeding 4, suggesting acceptable signal-to-noise ratios. Moreover, the design analysis (Table 2) showed close agreement between adjusted and predicted R^2^ values for all measured responses, confirming the reliability and robustness of the Design-Expert models.

### 3.2. Effect of Formulation Variables on the EE% (Y_1_)

**Figure 1:**
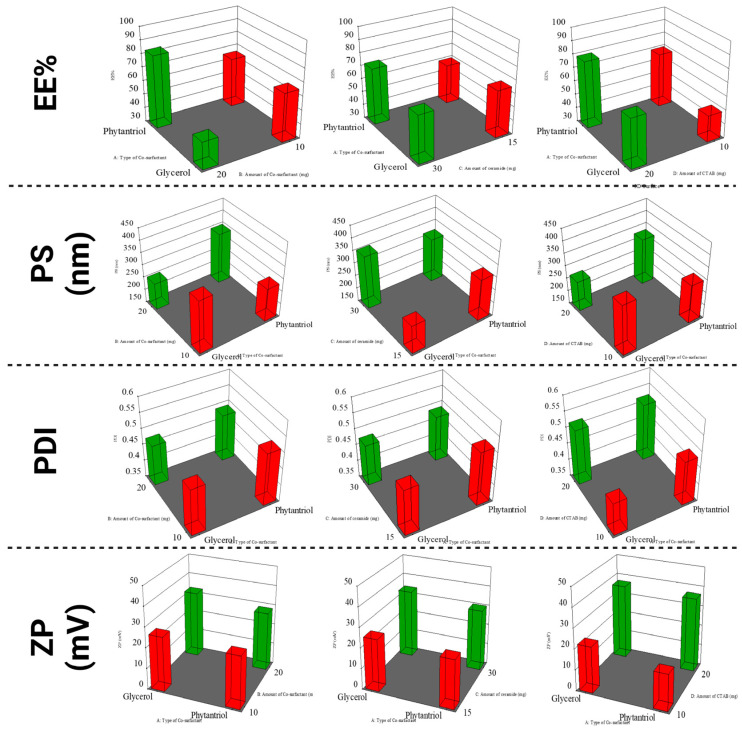
Influence of cosurfactant type and concentration on LOS-CER formulations.

The EE% of the formulated LOS-CERs ranged from 41.50 ± 0.39% to 97.07 ± 0.07%, and the drug LC% ranged from 18.20 ± 0.0004% to 53.60 ± 0.003%. (Table 3). Statistical analysis indicated no significant effect (*p* ≥ 0.05) on the LC% among the tested formulations. The relatively low LC percentages are typical for hydrophilic drugs, such as LOS, reflecting their limited affinity toward the lipid matrix. However, the consistent LC% across formulations confirms uniform drug incorporation within the vesicular systems. The highest EE% was observed in formulation F16, which contained phytantriol (20 mg), ceramide (30 mg), and CTAB (20 mg) at relatively high concentrations. These components are known to enhance the viscosity of vesicular dispersions [25]. Increased viscosity can hinder drug diffusion from the internal vesicular core to the external phase [26], thereby promoting greater retention of LOS within the vesicles and resulting in higher EE%. Statistical analysis demonstrated that all tested formulation variables significantly influenced EE% (Figure 1).

(1) Cosurfactant type (glycerol versus phytantriol):

Phytantriol-based formulations (red bars) consistently showed higher EE% than glycerol-based ones (green bars) under all formulation conditions (Table 3). This suggests that phytantriol enhances drug encapsulation, likely due to its more lipophilic nature (conferred by its phytanyl backbone, a long hydrophobic carbon chain that facilitates integration into CERs). Furthermore, phytantriol provides better bilayer structuring ability, which may strengthen drug-lipid interactions and thereby enhance encapsulation [27].

(2) Cosurfactant concentration:

As the concentration of cosurfactant increases from 10 to 30 mg, a moderate increase in EE% is observed, particularly in phytantriol-based formulations (Table 3). Higher cosurfactant concentrations may contribute to more organized vesicular structures and an expanded hydrophobic domain, favoring LOS encapsulation.

(3) Amount of ceramide:

Increasing the amount of ceramide generally resulted in an increase in EE%, especially when combined with phytantriol (Table 3). This suggests that higher ceramide content may stabilize the vesicle bilayer, promoting improved LOS encapsulation [8].

### 3.3. Effect of Formulation Variables on PS (Y_2_)

The PS of the formulated LOS-CERs ranged from 156.50 ± 0.50 nm to 442.50 ± 0.50 nm (Table 3). Statistical analysis demonstrated that all tested formulation variables significantly influenced PS (Figure 1).

(1) Type of cosurfactant (glycerol versus phytantriol):

Across all formulation conditions, phytantriol-based systems (red bars) consistently exhibited larger PS compared to glycerol-based ones (green bars) (Table 3). This suggests that phytantriol favors the formation of bulkier vesicles, likely due to its more hydrophobic, branched isoprenoid-like structure, and its tendency to form non-lamellar or highly ordered phases, both of which contribute to increase PS [27]. In turn, glycerol, being more hydrophilic, may promote the formation of smaller and more uniform vesicles [28].

(2) Amount of ceramide:

Increasing the ceramide concentration from 10 to 30 mg generally led to increase the PS, especially in phytantriol-based formulations (Table 3). This effect may be due to: (1) increased bilayer rigidity with higher ceramide content, resulting in stiffer and larger vesicles [8], and (2) an increased tendency for aggregation, as higher ceramide content can promote vesicle fusion or clustering, thereby enlarging the overall PS distribution [29].

### 3.4. Evaluation of the PDI

The PDI of the formulated LOS-CERs ranged from 0.42 ± 0.001 to 0.580 ± 0.001 (Table 3). These results indicate the relative homogeneity of the vesicles, which is consistent with previous literature [30]. Analysis of the formulation variables showed no significant effect on PDI values; therefore, PDI was excluded from the optimization process.

### 3.5. Effect of Formulation Variables on ZP (Y_3_)

The ZP of the formulated LOS-CERs ranged from +7.04 ± 0.04 mV to +47.36 ± 0.56 mV (Table 3). The positive ZP values could be related to the presence of quaternary ammonium group in the CTAB molecule [31]. Statistical analysis demonstrated that all tested formulation variables significantly influenced ZP (Figure 1).

(1) Type of cosurfactant (glycerol versus phytantriol):

Phytantriol-based formulations (red bars) showed lower ZP values compared to glycerol-based ones (green bars) across all formulation conditions (Table 3). This suggests that glycerol contributes to higher surface charge, possibly by altering the surface orientation or interaction of the lipid molecules, enhancing the electrostatic repulsion between vesicles [28]. The lower ZP of phytantriol-based formulations could indicate reduced repulsion, potentially increasing the risk of particle aggregation and precipitation over time.

(2) Amount of ceramide:

Increasing the ceramide concentration from 10 to 30 mg generally led to a slight increase in ZP, especially in glycerol-based formulations (Table 3). This effect may be due to enhanced lipid packing or surface modification caused by higher ceramide content, which can affect the surface charge distribution [8]. In turn, the effect was less pronounced in phytantriol-based formulations, possibly due to limited interactions between ceramide and the more rigid phytantriol bilayer structures.

### 3.6. Selection of the LOS-CERs

**Figure 2:**
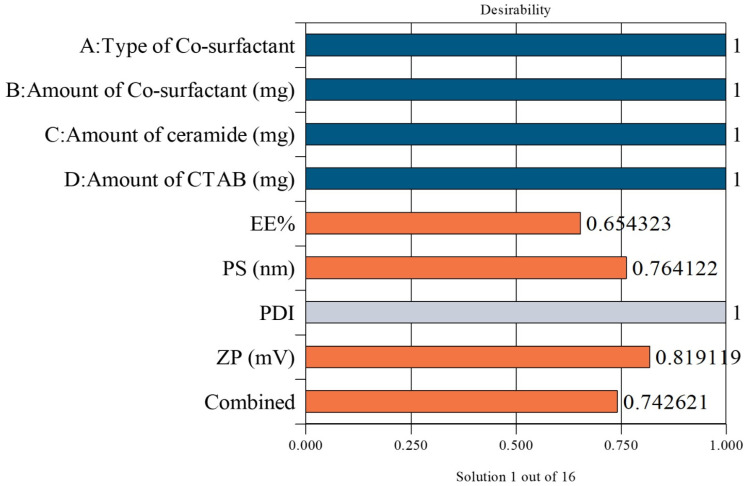
Desirability scores of individual factors and responses for the optimized LOS-CER formulation.

The optimized LOS-CER formulation was identified via Design-Expert^®^ software version 13 analysis of all prepared cerosomal formulations. The selected formulation demonstrated desirability scores of 0.654 for EE%, 0.764 for PS, 0.819 for ZP, resulting in an overall desirability score of 0.743, indicating a satisfactory balance among the targeted parameters (Figure 2). This optimized LOS-CER formulation was composed of 20 mg phytantriol, 30 mg ceramide, and 20 mg CTAB, and yielded an EE% of 97.07 ± 0.07%, a PS of 372.50 ± 0.50 nm, a PDI of 0.54 ± 0.02 mV, and a ZP of +33.24 ± 0.04 mV (Table 3). It is worth mentioning that the selected formulation, after CD functionalization, demonstrated an EE% of 98.00 ± 0.01%, a PS of 375.80 ± 1.20 nm, a PDI of 0.53 ± 0.01, and a ZP of +32.24 ± 0.01 mV. Statistical analysis revealed no significant differences (*p* ≥ 0.05) in these parameters following CD functionalization.

### 3.7. Fourier-Transform Infrared (FT-IR) Spectroscopy

**Figure 3:**
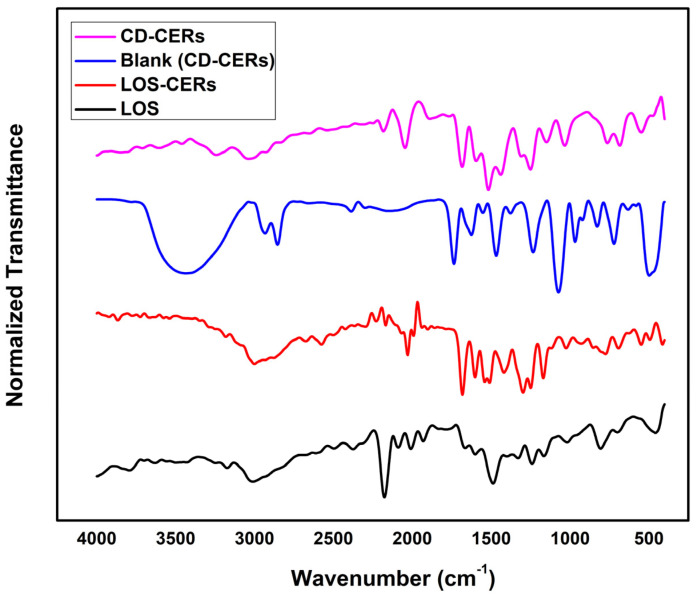
FT-IR spectra of LOS, LOS-CERs, CD-CERs, and Blank (CD-CERs).

Figure 3 shows the FT-IR spectra of LOS, LOS-CERs CD-CERs, and Blank (CD-CERs). The black spectrum of LOS exhibits characteristic peaks corresponding to its functional groups: a strong band at ~1740 cm^−1^ (C=O stretching of the carboxylic group), peaks at ~1600 cm^−1^ (aromatic C=C stretching), and bands in the 3100–2900 cm^−1^ range (C–H stretching vibrations) [32,33]. The red spectrum of LOS-CERs shows the characteristic lipid absorption peaks, including broad stretching in the 3400–3200 cm^−1^ region, corresponding to O–H/N–H stretching vibrations from lipid headgroups and residual hydration. Peaks around 2920–2850 cm^−1^ are due to C–H stretching of lipid alkyl chains. The band near 1650 cm^−1^ is characteristic of the carbonyl (C=O) stretching vibration of ester linkages, while peaks in the 1050–1150 cm^−1^ range indicate C–O–C stretching of phospholipid components. These bands confirm the structural integrity of the cerosomal vesicles [34,35]. The blue spectrum of Blank (CD-CERs) shows similar lipid-associated characteristics with slight broadening around 3400 cm^−1^, which is indicative of hydroxyl and amine groups from the CD surface and residual hydration. In the pink spectrum of the optimized CD-CER formulation, further broadening observed around ~3400 cm^−1^, indicating O–H and N–H groups on the CD surface. A distinct peak near ~1650 cm^−1^ is assigned to C=O stretching, while additional signals in the 1400–1000 cm^−1^ region correspond to C–O and C–N vibrations, confirming the successful surface functionalization of functional groups from CDs. Compared with LOS-CERs, CD-CERs exhibit shifts in the C=O and phospholipid-associated bands, suggesting hydrogen bonding and possible electrostatic interactions between the CDs and the phospholipid components of CERs. The appearance of both LOS and CD characteristic peaks in the CD-CER spectrum, along with noticeable shifts in the carbonyl and aromatic regions, indicates the successful loading of LOS and surface functionalization with CDs. These spectral modifications confirm intermolecular interactions among LOS, CDs, and the CER matrix, which may contribute to enhanced stability and drug-carrier compatibility.

### 3.8. Effect of Storage

At the end of the 3-month storage period, no noticeable changes were observed in the appearance of the optimized CD-CER formulation. Physicochemical evaluation of the stored formulation showed EE% of 95.00 ± 0.01%, PS of 382.50 ± 1.50 nm, PDI of 0.56 ± 0.01, and ZP of +32.00 ± 2.00 mV, with no significant differences compared to the freshly prepared formulation (*p* > 0.05). This good formulation stability may be attributed to the presence of CTAB, which likely enhances vesicle stability and membrane integrity by maintaining a highly positive ZP [16].

### 3.9. Morphological Evaluation by Transmission Electron Microscopy (TEM)

**Figure 4:**
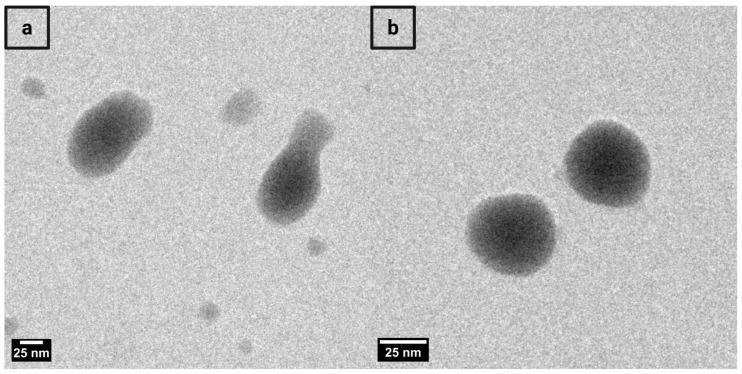
Transmission electron microscopy (TEM) images of (**a**) LOS-CERs and (**b**) CD-CERs, acquired at a magnification corresponding to a 25 nm scale bar.

The morphological characteristics of the optimized cerosomal formulation and its conjugated form were investigated using TEM, as shown in Figure 4 and Figures S2 and S3. Figure 4a displays the TEM micrograph of LOS-CERs, while Figure 4b represents the CD-CERs. The LOS-CERs appeared as small, variably shaped nanoparticles with some aggregation and a broader size distribution. In turn, the CD-CERs exhibited a more uniform, spherical morphology with well-dispersed particles and a narrower size distribution. The enhanced uniformity and dispersibility observed in CD-CERs may be attributed to the successful surface functionalization of CDs into the CER matrix, which potentially stabilizes the nanoparticle structure and improves overall homogeneity. These findings confirm the successful functionalization of CERs with CDs, as further supported by the FT-IR results indicating intermolecular interactions between CDs and cerosomal components.

It is noteworthy that the PS observed by TEM (25–50 nm) appeared considerably smaller than those measured by the Zetasizer (~380 nm). This discrepancy arises from the fundamental differences between the two techniques: the Zetasizer, based on dynamic light scattering (DLS), measures the hydrodynamic diameter, which includes the solvation and electrical double layers surrounding the hydrated nanoparticles, whereas TEM provides measurements under dry conditions, visualizing only the solid, dehydrated cores. Similar findings have been reported in previous studies, where hydrated-state measurements yielded larger apparent sizes compared to dehydrated TEM observations [36,37].

### 3.10. Drug Release Results

**Figure 5:**
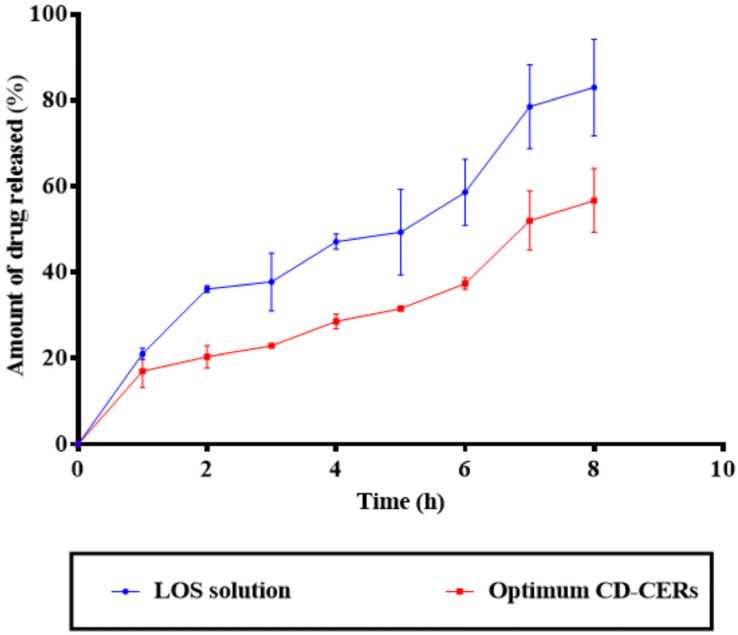
In vitro drug release profiles of LOS solution and optimum CD-CER formulation over 8 h.

As shown in Figure 5, the in vitro drug release profiles demonstrated a significant difference between the LOS solution and the optimized CD-CER formulation. The LOS solution exhibited rapid drug release, with over 82.99 ± 11.21% of the drug released within 8 h, characteristic of an immediate-release profile. In turn, the optimized CD-CER formulation showed a markedly slower and sustained drug release, with approximately 56.71 ± 7.36% released over the same period of time. The controlled-release profile of the optimized CD-CER formulation can be attributed to the structural characteristics of the CER matrix, which likely serves as a diffusion barrier, modulating the drug release rate and mechanism [8]. Such a controlled-release profile is desirable for maintaining consistent therapeutic levels, potentially reducing dosing frequency, and enhancing patient compliance [38]. Moreover, the relatively smaller standard deviations associated with the CD-CER formulation suggest improved reproducibility and formulation stability compared to the LOS solution.

### 3.11. Ex Vivo Permeation Results

**Figure 6:**
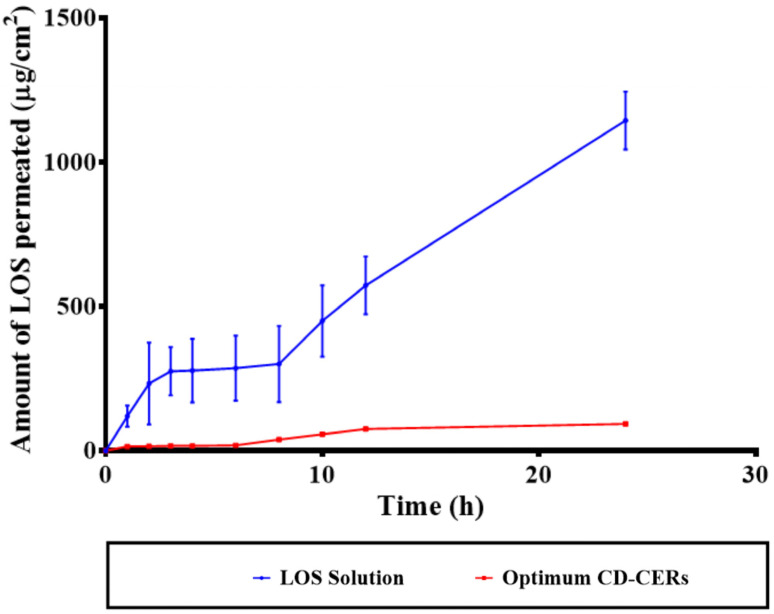
Ex vivo skin permeation profiles of LOS from LOS solution and optimum CD-CER formulation across excised rat skin over 24 h.

As shown in Figure 6, the ex vivo permeation study compared the transdermal delivery of LOS from the LOS solution and the optimized CD-CER formulation across excised rat skin. The LOS solution showed a markedly higher permeation rate, with the cumulative amount of LOS permeated reaching around 300.99 ± 85.87 µg/cm^2^ within 24 h. In turn, the optimized CD-CER formulation exhibited significantly lower permeation, with a small fraction of LOS crossing the skin barrier during the same period of time. This limited permeation is likely due to the controlled-release behavior of the CD-CER formulation, which retains the drug within the nanocarrier system for a prolonged time, minimizing burst release and systemic absorption [8]. Such a property is particularly advantageous for topical and localized transdermal applications, where sustained-release and site-specific drug action are desired [39]. These findings highlight the potential of CD-CERs to prolong drug retention at the site of action while reducing systemic exposure and associated adverse side effects. In addition, the ex vivo deposition results were 179.99 ± 20.99 µg/cm^2^ for the LOS solution and 420.98 ± 30.00 µg/cm^2^ for the CD-CERs, respectively.

### 3.12. Skin Penetration and Distribution Observed by Confocal Laser Scanning Microscopy (CLSM)

**Figure 7:**
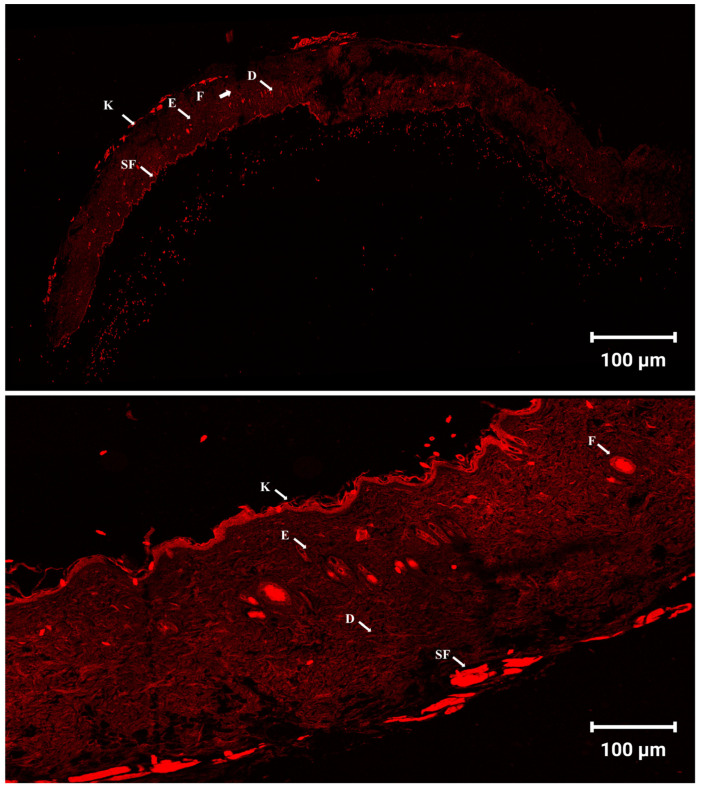
Tile scan confocal laser microscopy photomicrographs of longitudinal skin sections treated with the fluorophore-labeled optimized CD-CER formulation. The fluorescence intensity illustrates the penetration and distribution of the formulation across the different skin layers. Abbreviations: K, keratin; E, epidermis; D, dermis; F, follicles; and SF, subcutaneous fat.

The cellular uptake and skin permeation of the optimized CD-CER formulation were evaluated using CLSM. As shown in Figure 7 and Figure S4, strong red fluorescence was observed throughout the epidermal and dermal layers of the skin, indicating successful penetration of the fluorescently labeled optimized formulation. The distribution of the fluorescence signal beyond the superficial layers suggests effective transdermal delivery of the optimized formulation. These findings support the potential of CD-CERs as a promising hybrid vesicular drug delivery system for enhancing the therapeutic efficacy of repurposed drugs in the treatment of MRSA-associated skin infections. Notably, these results are consistent with those reported by Yang et al. [34], who successfully developed CERs for the delivery of methotrexate and nicotinamide in the treatment of skin psoriasis.

### 3.13. In Vivo Antibacterial Efficacy Against MRSA Skin Infection

**Figure 8:**
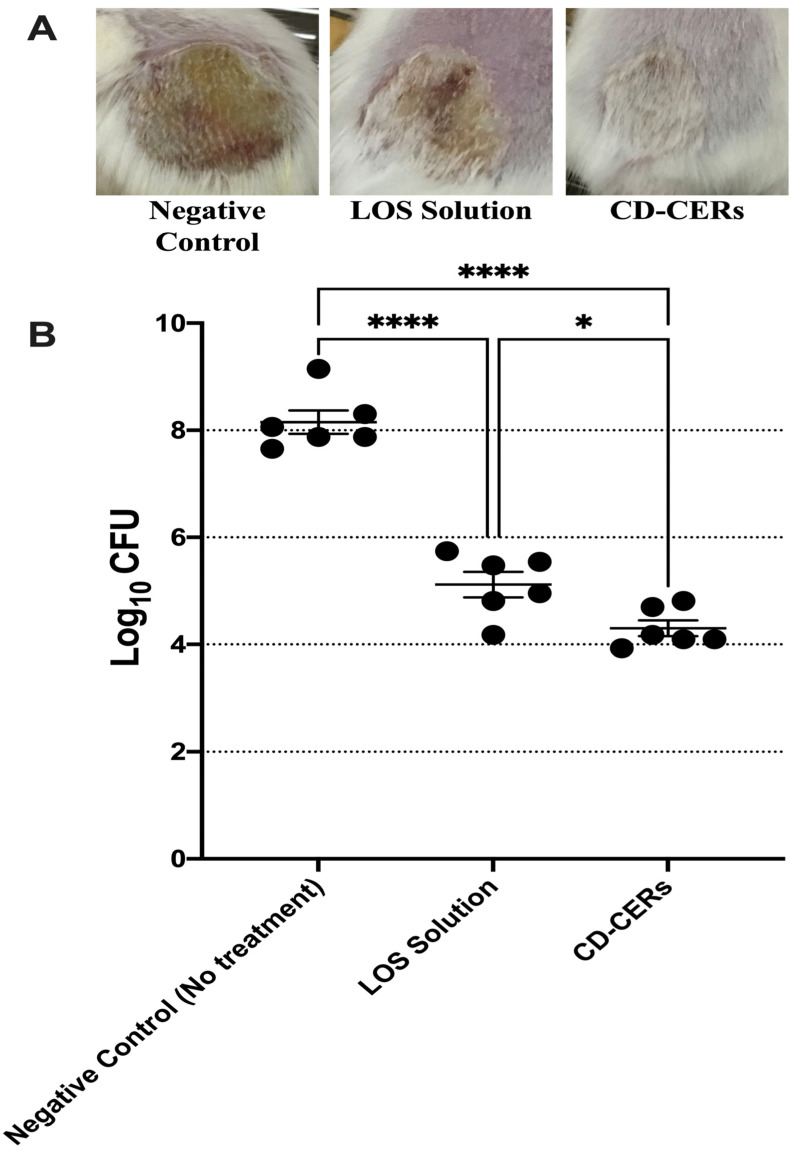
In vivo antibacterial efficacy of LOS solution and optimized CD-CER formulation in a murine MRSA skin infection model (*n* = 6 per group). (**A**) Representative dorsal photographs of mice showing treatment effects. (**B**) Quantitative bacterial loads from infected lesions, with each point representing an individual mouse. Data are presented as mean ± SEM. Statistical significance: * *p* < 0.05, **** *p* < 0.0001.

The in vivo antibacterial activity of the LOS solution and the optimized CD-CER formulation was evaluated using a murine MRSA USA300 skin infection model. Three groups of male BALB/c mice (*n* = 6 per group) were intradermally injected with a suspension of MRSA USA300. 48 h post-infection, abscesses developed at the injection sites, after which treatments were applied. As shown in Figure 8A, untreated control mice demonstrated severe abscesses with persistent erythema and swelling, whereas those treated with the LOS solution exhibited partial improvement. In turn, mice treated with the optimized CD-CER formulation showed marked reduction in lesion severity and grade, consistent with improved healing.

Quantitative bacterial counts further confirmed these observations (Figure 8B). Both the LOS solution and the optimized CD-CER formulation significantly reduced the bacterial count of MRSA USA300 compared to the untreated control group. For comparative assessment, the reference antibiotic vancomycin showed a minimum inhibitory concentration (MIC) of 1 µg/mL against MRSA USA300, as reported in our previous work, where losartan exhibited an MIC of 5 ± 0 mg/mL and an MBC of 10 ± 0 mg/mL against the same strain [13].

Moreover, the optimized CD-CER formulation demonstrated significantly greater antibacterial activity than the LOS solution. Specifically, treatment with the optimized CD-CER formulation reduced the bacterial load by 3.85 log_10_ CFU relative to the control, whereas the LOS solution achieved a 3.04 log_10_ CFU reduction. These findings confirm the superior in vivo antibacterial efficacy of the optimized CD-CER formulation compared to the free LOS solution, not only in terms of bacterial clearance but also in visible wound healing, highlighting its potential as a promising therapeutic strategy against drug-resistant skin infections.

### 3.14. Histopathological Evaluation of Skin Tissues

**Figure 9:**
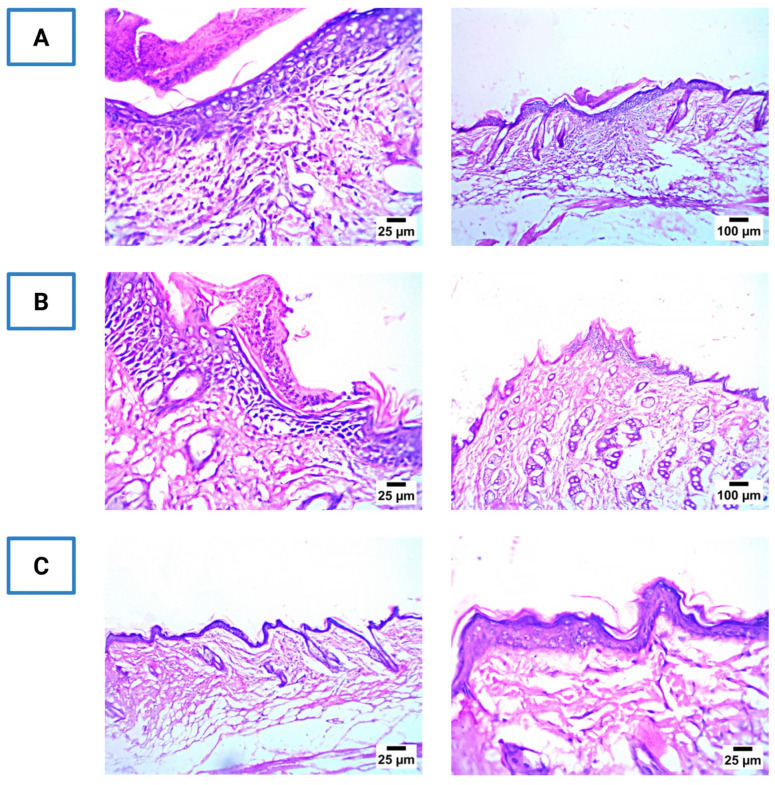
Representative histological micrographs of H&E-stained skin sections from MRSA-infected mice: (**A**) Control group; (**B**) LOS solution-treated; (**C**) CD-CER-treated.

The histopathological findings are presented in Figure 9. Skin sections from the untreated control group exhibited extensive necrosis, epidermal disruption, and dense inflammatory cell infiltration, indicating severe infection. The LOS solution-treated group showed partial epithelial regeneration and moderate inflammatory infiltration. In turn, the CD-CER-treated group demonstrated nearly complete re-epithelialization, restoration of normal dermal architecture, and minimal inflammation, suggesting significant wound healing and absence of tissue toxicity. These results confirm that CD-CERs not only enhance antibacterial efficacy but also promote skin repair without inducing adverse histological changes. These observations corroborate the antibacterial findings and confirm the safety of CD-CERs for topical application.

## 4. Conclusions

This study shows the successful development of losartan-loaded carbon dot-conjugated cerosomes (CD–CERs) as an advanced hybrid nanocarrier system for combating MRSA-associated skin infections. The optimized formulation demonstrated superior antibacterial activity, achieving greater bacterial clearance and improved healing outcomes compared with the free losartan solution. The synergistic combination of drug repurposing and nanocarrier functionalization enhanced therapeutic efficacy while sustaining localized drug release. These findings emphasize the translational potential of the developed CD–CERs as a promising therapeutic nanoplatform for the topical management of multidrug-resistant wound and implant infections, offering a foundation for future preclinical safety, mechanistic, and translational clinical studies.

## Figures and Tables

**Table 1:** Experimental Design and Responses Based on D-Optimal Mixture Design for LOS-Loaded CER Optimization.

Factors (Independent Variables)	Levels	
	Low (−1)	High (+1)
X_1_: Type of cosurfactant	Glycerol	Phytantriol
X_2_: Amount of cosurfactant (mg)	10	20
X_3_: Amount of ceramide (mg)	15	30
X_4_: Amount of CTAB (mg)	10	20
Responses (Dependent variables)	Constraints	
Y_1_: EE (%)	Maximize	
Y_2_: PS (nm)	Minimize	
Y_3_: ZP (mV)	Maximize	

Abbreviations: LOS, Losartan potassium; CERs, Cerosomes; CTAB, Cetyltrimethylammonium bromide; EE%, Entrapment efficiency; PS, Particle size; and ZP, Zeta potential.

**Table 2:** Predicted and observed responses of LOS-CER formulations from D-optimal design.

Responses	R^2^	Adjusted R^2^	Predicted R^2^	Adequate Precision	Significant Factors
EE%	0.999	0.999	0.998	330.835	X_1_, X_2_, X_3_, X_4_
PS (nm)	1	0.999	0.999	730.89	X_1_, X_2_, X_3_, X_4_
ZP (mV)	0.893	0.842	0.758	12.87	X_2_, X_3_, X_4_
	EE%	PS (nm)	PDI	ZP (mV)	
Predicted values for the selected LOS-CERs	76.90	223.69	0.517	+40.15	
Observed values for the selected LOS-CERs	77.11	223.50	0.52	+45.95	

Abbreviations: LOS, Losartan potassium; CERs, Cerosomes; EE%, Entrapment efficiency; PS, Particle size; PDI, Polydispersity index; and ZP, Zeta potential.

**Table 3:** Experimental runs and measured responses of LOS-CER formulations.

Formulation Code	Type of Co-Surfactant (X_1_)	Amount of Cosurfactant (mg) (X_2_)	Amount of Ceramide (mg) (X_3_)	Amount of CTAB (mg) (X_4_)	EE (%)	LC (%)	PS (nm)	PDI	ZP (mV)
F1	Glycerol	10	30	10	58.82 ± 0.12	29.20 ± 0.0002	442.50 ± 0.50	0.421 ± 0.013	+22.80 ± 0.20
F2	Glycerol	10	15	10	60.67 ± 0.02	31.20 ± 0.001	357.50 ± 0.50	0.470 ± 0.001	+16.40 ± 0.10
F3	Glycerol	10	15	20	71.80 ± 0.20	53.60 ± 0.003	369.50 ± 0.50	0.580 ± 0.001	+33.39 ± 0.29
F4	Glycerol	10	30	20	73.45 ± 0.45	34.30 ± 0.001	291.50 ± 0.50	0.520 ± 0.01	+35.50 ± 0.50
F5	Glycerol	20	30	10	38.80 ± 0.20	18.20 ± 0.0004	442.50 ± 0.50	0.480 ± 0.03	+31.68 ± 0.48
F6	Glycerol	20	15	10	41.50 ± 0.39	21.60 ± 0.001	170.50 ± 0.50	0.440 ± 0.05	+20.90 ± 0.10
F7	Glycerol	20	15	20	59.36 ± 0.36	31.40 ± 0.002	156.50 ± 0.50	0.490 ± 0.01	+33.40 ± 0.10
F8	Glycerol	20	30	20	60.94 ± 0.06	27.10 ± 0.002	265.50 ± 0.50	0.500 ± 0.04	+47.36 ± 0.56
F9	Phytantriol	10	15	10	59.15 ± 0.04	44.70 ± 0.003	267.50 ± 0.50	0.54 ± 0.001	+7.04 ± 0.04
F10	Phytantriol	10	15	20	62.25 ± 0.25	32.40 ± 0.001	392.50 ± 0.50	0.51 ± 0.01	+30.83 ± 0.13
F11	Phytantriol	10	30	10	69.35 ± 0.35	34.80 ± 0.001	262.00 ± 1.00	0.500 ± 0.04	+19.00 ± 0.70
F12	Phytantriol	10	30	20	77.11 ± 0.10	36.40 ± 0.002	223.50 ± 0.50	0.52 ± 0.01	+45.95 ± 0.45
F13	Phytantriol	20	15	10	72.55 ± 0.50	37.60 ± 0.001	254.50 ± 0.50	0.490 ± 0.01	+21.05 ± 0.45
F14	Phytantriol	20	30	10	82.30 ± 0.30	39.00 ± 0.001	436.50 ± 0.50	0.42 ± 0.001	+24.63 ± 0.53
F15	Phytantriol	20	15	20	83.01 ± 0.01	40.40 ± 0.002	353.85 ± 0.05	0.54 ± 0.05	+37.83 ± 1.33
F16	Phytantriol	20	30	20	97.07 ± 0.07	43.00 ± 0.002	372.50 ± 0.50	0.54 ± 0.02	+33.24 ± 0.04

## Data Availability

The original contributions presented in the study are included in the article, further inquiries can be directed to the corresponding authors.

## Supplementary Materials

The following supporting information can be downloaded at: https://www.mdpi.com/article/10.3390/pharmaceutics17111483/s1, Figure S1. The UV–Vis absorption spectrum of the prepared CDs; Figure S2. Transmission electron microscopy (TEM) images of the optimum LOS-CERs formulation at magnifications corresponding to 100 nm, 50 nm, and 25 nm scale bars; Figure S3. Transmission electron microscopy (TEM) images of the optimum CD-CERs formulation at magnifications corresponding to 50 nm and 25 nm scale bars; Figure S4. Tile scan confocal laser microscopy.

## References

[1] Tahmasebi H., Arjmand N., Monemi M., Babaeizad A., Alibabaei F., Alibabaei N., Bahar A., Oksenych V., Eslami M. (2025). From cure to crisis: Understanding the evolution of antibiotic-resistant bacteria in human microbiota. Biomolecules.

[2] Lin Y., Liu L., He J., Shen J., Ren Q. (2025). Rapid release of high-valent silver ions from water-soluble porphyrin complexes to enhance the direct killing of Methicillin-Resistant Staphylococcus aureus. Acta Biomater..

[3] Lei D., Dong X., Yang T., Jin Y., Zhou W. (2025). Clade-specific adaptation and global spread of *Staphylococcus aureus* ST188 with emergence of a multidrug-resistant MRSA sublineage. Msystems.

[4] Konreddy A.K., Rani G.U., Lee K., Choi Y. (2019). Recent drug-repurposing-driven advances in the discovery of novel antibiotics. Curr. Med. Chem..

[5] Lehar S.M., Pillow T., Xu M., Staben L., Kajihara K.K., Vandlen R., DePalatis L., Raab H., Hazenbos W.L., Morisaki J.H. (2015). Novel antibody–antibiotic conjugate eliminates intracellular *S. aureus*. Nature.

[6] Pelgrift R.Y., Friedman A.J. (2013). Nanotechnology as a therapeutic tool to combat microbial resistance. Adv. Drug Deliv. Rev..

[7] Kahraman E., Kaykın M., Şahin Bektay H., Güngör S. (2019). Recent advances on topical application of ceramides to restore barrier function of skin. Cosmetics.

[8] Gaur P.K., Purohit S., Kumar Y., Mishra S., Bhandari A. (2014). Ceramide-2 nanovesicles for effective transdermal delivery: Development, characterization and pharmacokinetic evaluation. Drug Dev. Ind. Pharm..

[9] Oliveira E.G.d.L., de Oliveira H.P., Gomes A.S.L. (2020). Metal nanoparticles/carbon dots nanocomposites for SERS devices: Trends and perspectives. SN Appl. Sci..

[10] Mou C., Wang X., Teng J., Xie Z., Zheng M. (2022). Injectable self-healing hydrogel fabricated from antibacterial carbon dots and ɛ-polylysine for promoting bacteria-infected wound healing. J. Nanobiotechnol..

[11] Lim S.Y., Shen W., Gao Z. (2015). Carbon quantum dots and their applications. Chem. Soc. Rev..

[12] Thakur M., Pandey S., Mewada A., Patil V., Khade M., Goshi E., Sharon M. (2014). Antibiotic conjugated fluorescent carbon dots as a theranostic agent for controlled drug release, bioimaging, and enhanced antimicrobial activity. J. Drug Deliv..

[13] Albash R., Hassan M., Agiba A.M., Mohamed H.W., Hassan M.S., Ali R.M., Shalabi Y.E., Omran H.M.A., Eltabeeb M.A., Alamoudi J.A. (2025). Advanced Vaginal Nanodelivery of Losartan Potassium via PEGylated Zein Nanoparticles for Methicillin-Resistant Staphylococcus aureus. Pharmaceutics.

[14] Ahmed S., Aziz D.E., Sadek M.A., Tawfik M.A. (2024). Capped flexosomes for prominent anti-inflammatory activity: Development, optimization, and ex vivo and in vivo assessments. Drug Deliv. Transl. Res..

[15] Albash R., Ali S.K., Abdelmonem R., Agiba A.M., Aldhahri R., Saleh A., Kassem A.B., Abdellatif M.M. (2025). Electrospun nanofiber-scaffold-loaded levocetirizine dihydrochloride cerosomes for combined management of atopic dermatitis and methicillin-resistant Staphylococcus Aureus (MRSA) skin infection: In vitro and in vivo studies. Pharmaceuticals.

[16] Agiba A.M., Rodríguez Huerta L.G., Ulloa-Castillo N.A., Sierra-Valdez F.J., Beigi-Boroujeni S., Lozano O., Aguirre-Soto A. (2025). Fusion of polymer-coated liposomes and centrifugally spun microfibers as hybrid materials to enhance sustained release. Nanoscale Adv..

[17] Seedad R., Ratanawimarnwong N., Jittangprasert P., Mantim T., Songsrirote K. (2021). Carbon dots prepared from citric acid and urea by microwave-assisted irradiation as a turn-on Carbon fluorescent probe for allantoin determination. New J. Chem..

[18] Alzahrani A., Alsulami T., Salamatullah A.M., Ahmed S.R. (2023). Non-spherical gold nanoparticles enhanced fluorescence of carbon dots for norovirus-like particles detection. J. Biol. Eng..

[19] Albash R., Abdelbari M.A., Elbesh R.M., Khaleel E.F., Badi R.M., Eldehna W.M., Elkaeed E.B., El Hassab M.A., Ahmed S.M., Mosallam S. (2024). Sonophoresis mediated diffusion of caffeine loaded Transcutol® enriched cerosomes for topical management of cellulite. Eur. J. Pharm. Sci..

[20] National Research Council (2011). Guide for the Care and Use of Laboratory Animals.

[21] Eita A.S., Makky A.M., Anter A., Khalil I.A. (2025). Foamable pluroleosomes system loaded with amlodipine as a repurposed antibacterial topical formulation against MRSA-induced infection; optimization, in-vitro, ex-vivo, and in-vivo studies. Int. J. Pharm. X.

[22] Mohamed S.A., Eraqi W.A., Georghiou P.E., Zakaria M.Y. (2025). Luteolin loaded PEGylated cerosomes: A novel treatment for MRSA skin infections. BMC Microbiol..

[23] Bancroft J.D., Gamble M. (2008). Theory and Practice of Histological Techniques.

[24] Yu L.X. (2008). Pharmaceutical quality by design: Product and process development, understanding, and control. Pharm. Res..

[25] Rowe R.C., Sheskey P.J., Owen S.C. (2009). Handbook of Pharmaceutical Excipients.

[26] Patel H., Kundawala A., Patel N. (2009). Effect of lipid and surfactant concentration on physicochemical properties and entrapment efficiency of niosomes prepared for topical delivery of erythromycin. Int. J. Drug Deliv. Technol..

[27] Boyd B.J., Khoo S.M., Whittaker D.V., Davey G., Porter C.J.H. (2009). Nanostructured lipid dispersions formed from phytantriol—Templating of cubic lyotropic liquid crystalline phases. Int. J. Pharm..

[28] Abdallah M.H., Shahien M.M., El-Horany H.E.S., Ahmed E.H. (2025). Modified Phospholipid Vesicular Gel for Transdermal Drug Delivery: The Influence of Glycerin and/or Ethanol on Their Lipid Bilayer Fluidity and Penetration Characteristics. Gels.

[29] Imura T., Sakai H., Yamauchi H., Kaise C., Kozawa K., Yokoyama S., Abe M. (2001). Preparation of liposomes containing Ceramide 3 and their membrane characteristics. Colloids Surf. B Biointerfaces.

[30] El Hassab M.A., Ibrahim M.H., Abdel Mageed S.S., Mahmoud A.M.A., Othman Ahmed Z.S., Mosallam S., Abdelbari M.A., Khaleel E.F., El Hasab M.A., Elsayyad A. (2025). Correction: Formulation of zein nanoparticles for augmenting the anti-inflammatory activity of dexketoprofen. Front. Pharmacol..

[31] Tawfik M.A., Ahmed S., El-Dahmy R.M., Aziz D.E. (2025). Oleic acid Enriched Leciplexes as Novel Mucoadhesive Cationic Nanocarriers of Agomelatine for Glaucoma Treatment. AAPS PharmSciTech.

[32] Al-Majed A.-R.A., Assiri E., Khalil N.Y., Abdel-Aziz H.A. (2015). Losartan: Comprehensive profile. Profiles Drug Subst. Excip. Relat. Methodol..

[33] Paraschiv M., Smaranda I., Zgura I., Ganea P., Chivu M., Chiricuta B., Baibarac M. (2022). Degradation of losartan potassium highlighted by correlated studies of photoluminescence, infrared absorption spectroscopy and dielectric spectroscopy. Pharmaceutics.

[34] Yang X., Tang Y., Wang M., Wang Y., Wang W., Pang M., Xu Y. (2021). Co-delivery of methotrexate and nicotinamide by cerosomes for topical psoriasis treatment with enhanced efficacy. Int. J. Pharm..

[35] Elhabal S.F., Abdelaal N., Al-Zuhairy S.A.S., Mohamed Elrefai M.F., Khalifa M.M., Khasawneh M.A., Elsaid Hamdan A.M., Mohie P.M., Gad R.A., Kabil S.L. (2024). Revolutionizing psoriasis topical treatment: Enhanced efficacy through ceramide/phospholipid composite cerosomes co-delivery of cyclosporine and dithranol: In-vitro, ex-vivo, and in-vivo studies. Int. J. Nanomed..

[36] Prabha S., Zhou W.Z., Panyam J., Labhasetwar V. (2002). Size-dependency of nanoparticle-mediated gene transfection: Studies with fractionated nanoparticles. Int. J. Pharm..

[37] Morsi N., Ghorab D., Refai H., Teba H. (2016). Ketoroloac tromethamine loaded nanodispersion incorporated into thermosensitive in situ gel for prolonged ocular delivery. Int. J. Pharm..

[38] Ahmed S., Ibrahim M.M., Balkhi B., Attia H., Aziz D.E. (2025). From Bench to Biology: Unraveling the Efficiency of novel Brijosomes for Trans-Tympanic Drug Delivery. J. Drug Deliv. Sci. Technol..

[39] Prausnitz M.R., Langer R. (2008). Transdermal drug delivery. Nat. Biotechnol..

