# Supplementary material for: Overcoming MRSA Antibiotic Resistance Through Losartan Repurposing with Carbon Dot–Conjugated Cerosomal Nanocarriers
Source: Pharmaceutics. 2025 Nov 17;17(11):1483. doi: 10.3390/pharmaceutics17111483 (PMC12655805; doi:10.3390/pharmaceutics17111483)
Supplement: Supplementary file 1 [file pharmaceutics-17-01483-s001.zip › pharmaceutics-3939331-supplementary.pdf]

Overcoming MRSA Antibiotic Resistance Through Losartan Repurposing with Carbon Dot–Conjugated Cerosomal Nanocarriers

Yasmina Elmahboub [a], Rofida Albash [a,*], Ahmed M. Agiba [b,], Mariam Hassan [c,d], Haneen Waleed Mohamed [e], Mohamed Safwat Hassan [e], Roaa Mohamed Ali [e], Yara E. Shalabi [e], Hend Mahmoud Abdelaziz Omran [e], Ahmed Adel Alaa-Eldin [f], Jawaher Abdullah Alamoudi [g], Asmaa Saleh [g], Amira B. Kassem [h] and Moaz A. Eltabeeb [i]**

[a] Department of Pharmaceutics, College of Pharmaceutical Sciences and Drug Manufacturing, Misr University for Science and Technology, Giza 12585, Egypt
[b] School of Engineering and Sciences, Tecnologico de Monterrey, Monterrey 64849, Mexico
[c] Department of Microbiology and Immunology, Faculty of Pharmacy Cairo University, Cairo 11562, Egypt
[d] Department of Microbiology and Immunology, Faculty of Pharmacy, Galala University, New Galala City, Suez 43511, Egypt
[e] College of Pharmaceutical Sciences and Drug Manufacturing, Misr University for Science and Technology, Giza 12585, Egypt
[f] Department of Pharmaceutics, Faculty of Pharmacy, Fayoum University, Faiyum 2933051, Egypt
[g] Department of Pharmaceutical Sciences, College of Pharmacy, Princess Nourah Bint Abdulrahman University, P.O. Box 84428, Riyadh 11671, Saudi Arabia
[h] Clinical Pharmacy and Pharmacy Practice Department, Faculty of Pharmacy, Damanhour University, Damanhour 22514, Egypt
[I] Department of Industrial Pharmacy, College of Pharmaceutical Sciences and Drug Manufacturing, Misr University for Science and Technology, Giza 12585, Egypt
Correspondence: Rofida Albash* (rofida.albash@must.edu) and Ahmed M. Agiba (ahmed.agiba@tec.mx).**

Supplementary Information

<u>Supplementary Figures</u>

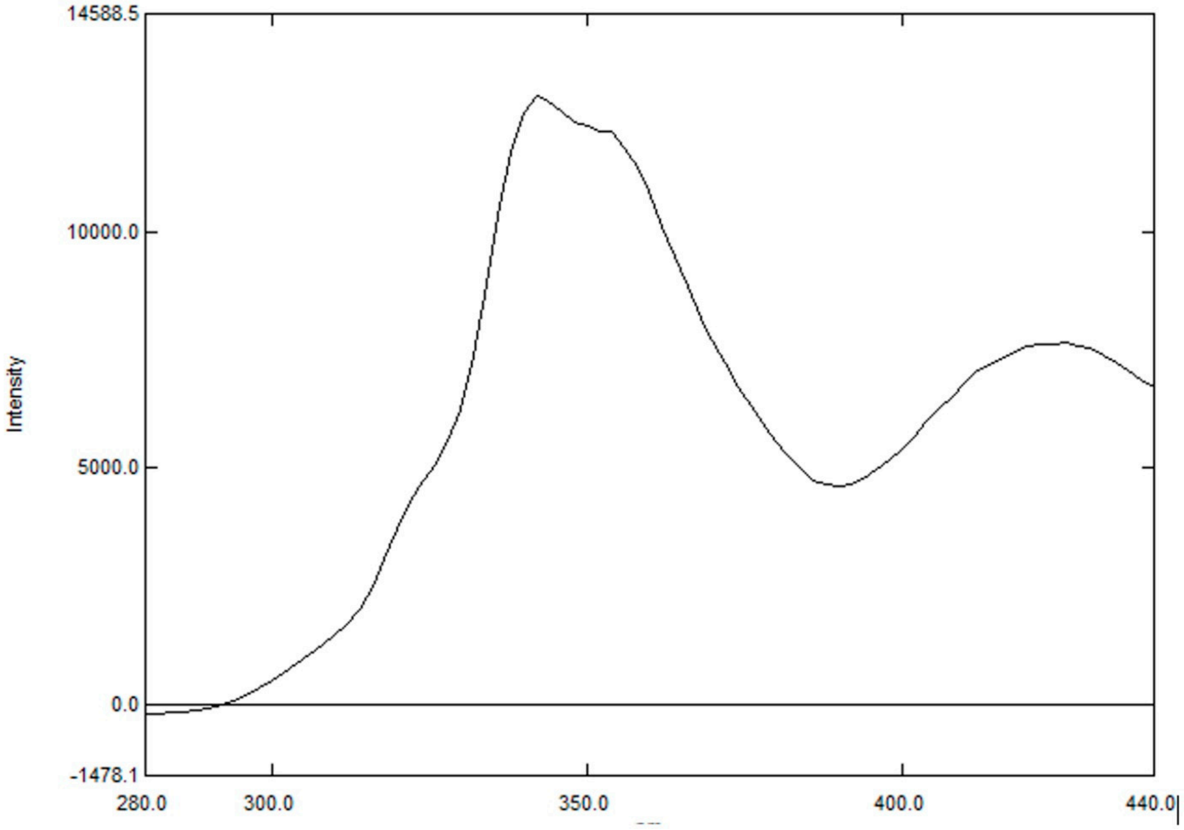

Figure S1. The UV–Vis absorption spectrum of the prepared CDs.

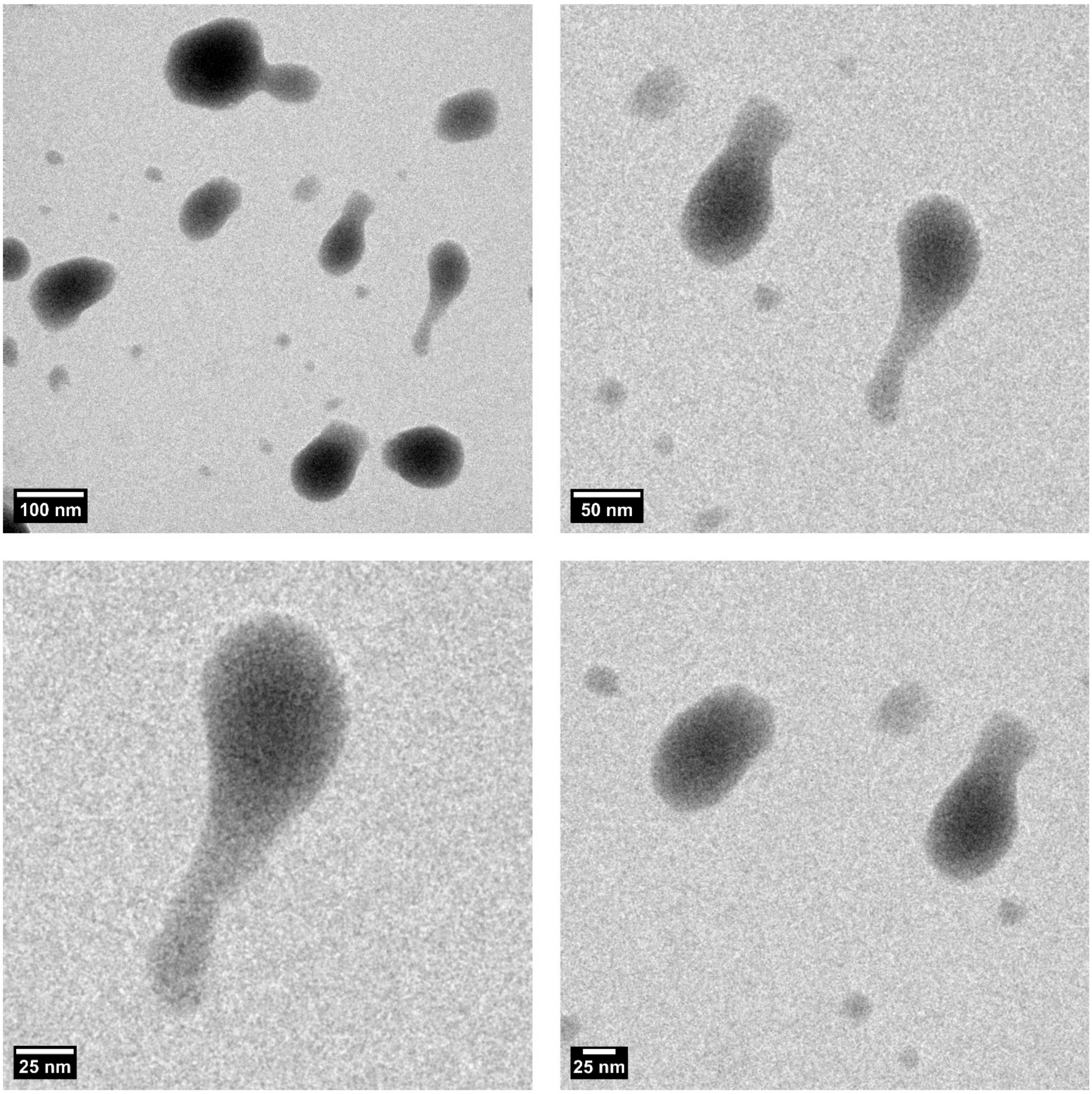

Figure S2. Transmission electron microscopy (TEM) images of the optimum LOS-CERs formulation at magnifications corresponding to 100 nm, 50 nm, and 25 nm scale bars.

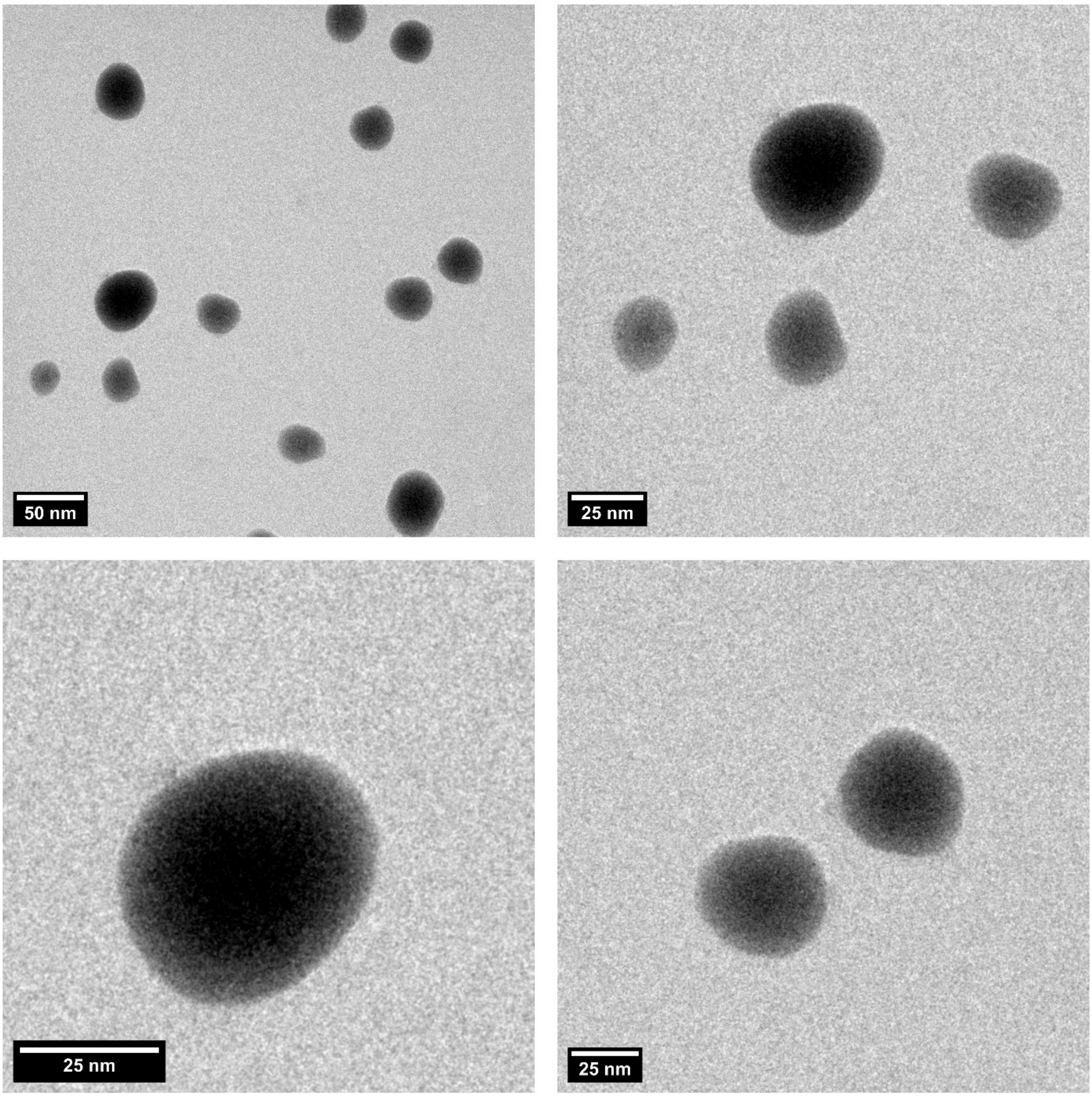

Figure S3. Transmission electron microscopy (TEM) images of the optimum CD-CERs formulation at magnifications corresponding to 50 nm and 25 nm scale bars.

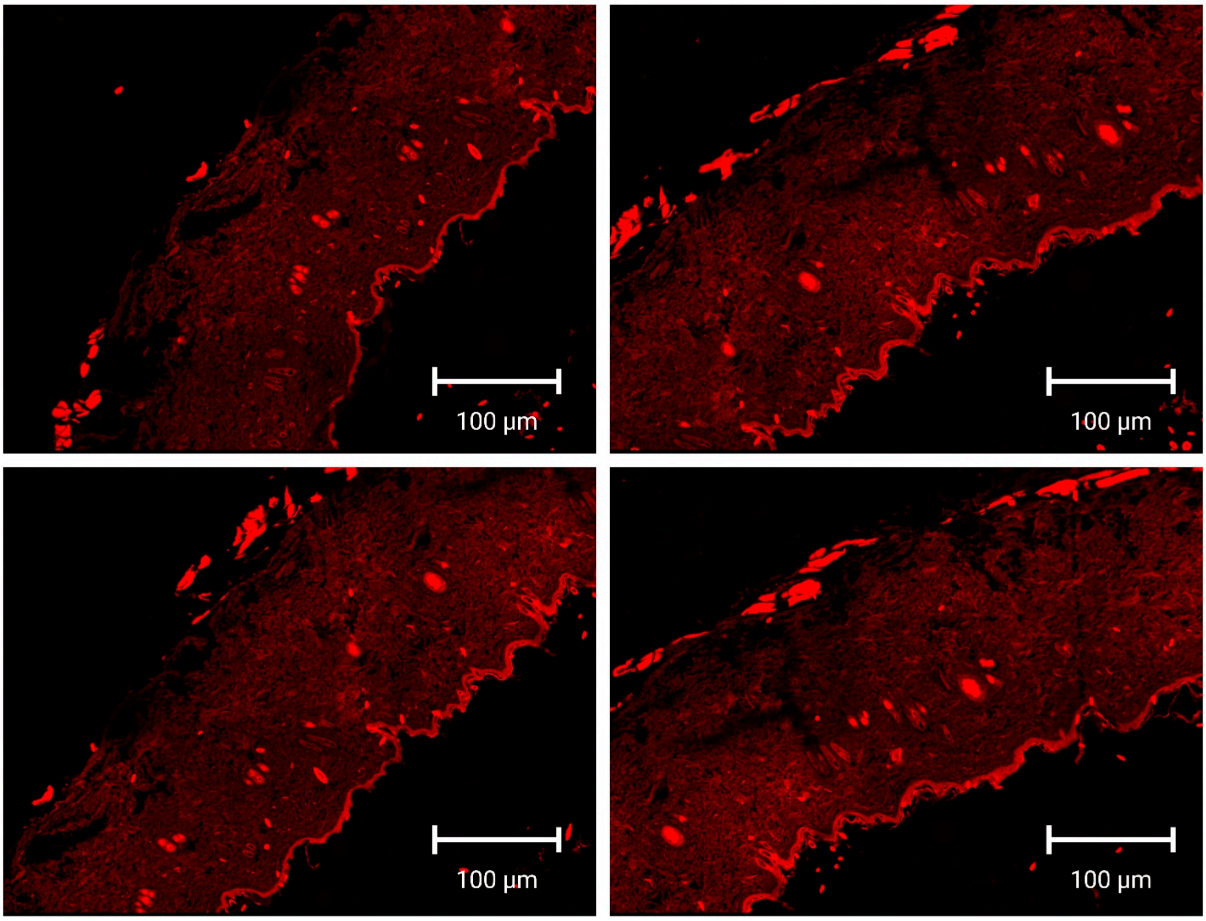

Figure S4. Tile scan confocal laser microscopy photomicrographs of longitudinal skin sections treated with the fluorophore-labeled optimized CD-CERs formulation.
